# Reelin Signaling Inactivates Cofilin to Stabilize the Cytoskeleton of Migrating Cortical Neurons

**DOI:** 10.3389/fncel.2017.00148

**Published:** 2017-05-23

**Authors:** Michael Frotscher, Shanting Zhao, Shaobo Wang, Xuejun Chai

**Affiliations:** ^1^Center for Molecular Neurobiology Hamburg (ZMNH), Institute for Structural Neurobiology, University Medical Center Hamburg-EppendorfHamburg, Germany; ^2^College of Veterinary Medicine, Northwest A&F UniversityYangling, China

**Keywords:** neuronal orientation, cortical pyramidal neuron, neuronal migration, leading process, trailing process, Reelin, cofilin, *in utero* electroporation

## Abstract

Neurons are highly polarized cells. They give rise to several dendrites but only one axon. In addition, many neurons show a preferred orientation. For example, pyramidal neurons of the cerebral cortex extend their apical dendrites toward the cortical surface while their axons run in opposite direction toward the white matter. This characteristic orientation reflects the migratory trajectory of a pyramidal cell during cortical development: the leading process (the future apical dendrite) extends toward the marginal zone (MZ) and the trailing process (the future axon) toward the intermediate zone (IZ) while the cells migrate radially to reach their destination in the cortical plate (CP). In this review article, we summarize the function of Reelin, an extracellular matrix protein synthesized by Cajal-Retzius cells in the MZ, in the development of the characteristic orientation of the leading processes running perpendicular to the cortical surface. Reelin promotes migration toward the cortical surface since late-generated cortical neurons in the *reeler* mutant are unable to reach upper cortical layers. Likewise, Reelin is important for the orientation and maintenance of the leading processes of migrating neurons since they are misoriented in the developing *reeler* cortex, as are the apical dendrites of pyramidal cells in the mature mutant. Reelin-induced phosphorylation of cofilin, an actin-associated protein, is crucial since pyramidal neurons transfected by *in utero* electroporation (IUE) with a non-phosphorylatable form of cofilin (*cofilin*^S3A^) show severe migration defects reminiscent of those in the *reeler* mutant. Remarkably, migration of neurons in the cortex of *reeler* mice was partially rescued by transfecting them with *LIM kinase 1* (*LIMK1*), the kinase that induces phosphorylation of cofilin at serine3, or with a pseudo-phosphorylated cofilin mutant (*cofilin*^S3E^). Together these results indicate that Reelin-induced phosphorylation of cofilin is an important component in the orientation and directed migration of cortical neurons and in their correct lamination.

## Introduction

The student of the mammalian cerebral cortex is impressed by two phenomena pointing to a well-ordered, non-random structural organization. First, there is the arrangement of cortical neurons in layers as seen in Nissl-stained sections or in sections immunostained using layer specific markers. Second, there is an almost uniform vertical orientation of the vast majority of cortical neurons. This is nicely seen when cortical neurons are impregnated using the Golgi method which stains the cells with their axonal and dendritic processes (Golgi, 1873). The vertical orientation mainly comes about by the thick, long apical dendrites originating from the cell bodies and running toward the pial surface where they branch, forming apical tufts. From the opposite poles of the cell bodies the axons originate, which run toward the white matter. Thus, this vertical orientation of virtually all cortical pyramidal neurons nicely illustrates the characteristic bipolarity of nerve cells having one efferent process, the axon, and many dendrites being the receptive processes of the cell conveying incoming signals toward the cell body. In a cortical pyramidal cell the two processes, the apical dendrite and the axon, run in fact in opposite directions as noticed early on by investigators applying the Golgi method (e.g., Ramón y Cajal, 1911).

From a cell biological point of view numerous questions arise after this short description of cortical organization. Numerous researchers have dealt intensely with these questions during the last decades. In a landmark article, Berry and Rogers (1965) described for the first time that there is an “inside-out” lamination with early-generated neurons establishing the deep layers and late-generated neurons the superficial layers of the cerebral cortex. In a way, this finding came as a surprise because the late-generated neurons did not simply push away the early-generated ones, which would have resulted in an opposite layering. Late-generated neurons rather have to tackle the task of bypassing a densely packed layer of earlier generated cells. This also includes that late-generated neurons trespassing early-generated deep layer cells have to migrate for much longer distances to reach their destination. How is this achieved? How do late-generated neurons find their way to the surface of the cortex?

Developmental studies in various species and more recently real-time microscopy of migrating neurons in the cortex have contributed much to our understanding of the journey of late-generated neurons through the layers of their predecessors (Nadarajah et al., 2001; Nadarajah and Parnavelas, 2002). It has become clear that a migrating neuron moves in the direction of its thick leading process and that this is associated with a translocation of the nucleus in this direction. With the leading process becoming the main apical dendrite, the vertical orientation of pyramidal cells in the mature cortex represents a frozen picture of its development, particularly of the directionality of the migratory process. What are the signals that control this directionality? Radial glial cell processes span the distance between the ventricle and the cortical surface, serving as a guiding scaffold for migrating neurons (Rakic, 1972). What are the signals controlling their orientation? What are the signals that anchor the leading processes of pyramidal neurons to the pial surface and how is this particular vertical orientation of apical dendrites maintained throughout life?

Much has been learned in the past from mouse mutants. A perfect example is the *reeler* mutant described as early as in 1951 (Falconer, 1951). In this mutant, the normal inside-out lamination of the cerebral cortex is inverted and the majority of cortical pyramidal cells are misoriented, pointing to an important defect in molecular pathways but at the same time also to an interdependence of process orientation and cortical layer formation. With the discovery of Reelin, the molecule deficient in the *reeler* mutant, research in this area has exploded and our understanding of layer formation in the cortex and of the different players involved has significantly improved.

## Reelin Receptors and The Reelin Signaling Cascade

The name Reelin is attributed to *reeler*, a natural mouse mutant described long before the Reelin gene was found (Falconer, 1951). As suggested by its name, the *reeler* mouse shows an impressive phenotype dominated by severe ataxia. Reelin is a large 400 kDa glycoprotein of the extracellular matrix which is synthesized and secreted by Cajal-Retzius cells (D’Arcangelo et al., 1995; Ogawa et al., 1995), an early class of cortical neurons that were described in detail in Golgi preparations by Santiago Rámon y Cajal and Gustaf Retzius at the turn of last century. Of note, Cajal-Retzius cells populate the marginal zone (MZ) of the developing cortex, future layer I. Thus, full-length Reelin and its fragments are highly concentrated at the top of the developing cortex, suggesting that this characteristic topographical distribution is important for the control of migratory processes underneath, i.e., for the migration of cortical neurons from the ventricle to the cortical surface. Is Reelin in the MZ an attractive molecule for newborn neurons generated near the ventricle? Nichols and Olson (2010) have shown that Reelin induces orientation and alignment of early-generated layer Vl neurons and preplate splitting. Moreover, a role for Reelin in the directed migration of cortical neurons is consistent with the observation that in *reeler* late-generated neurons are unable to migrate through deep, early-generated cell layers, likely because their attractive force is missing in the mutant (Zhao et al., 2004). However, in *reeler*, but not in wild type, layer I of the cortex, the former MZ, is densely populated, suggesting an opposite effect, a stop signal function for Reelin (Zhao and Frotscher, 2010). Could it be that full-length Reelin in the MZ has different functions than the various Reelin fragments, diffusing to deeper cortical layers? Reelin is composed of an N-terminal F-spondin-like domain, eight repeats, and a short and highly basic C-terminal region. Reelin is cleaved at two sites approximately located between repeats 2 and 3 and between repeats 6 and 7, resulting in a total of five different fragments (370 kDa, 270 kDa, 190 kDa, 180 kDa and 80 kDa) in addition to the full-length protein (Lambert de Rouvroit et al., 1999; Ignatova et al., 2004). Of note, inhibition of processing alters cortical lamination (unpublished observations).

Or could it be that different Reelin receptors were involved? It came as a surprise that Reelin binds to lipoprotein receptors and that a double knockout mouse deficient in the two lipoprotein receptors, apolipoprotein E receptor 2 (ApoER2) and very low-density lipoprotein receptor (VLDLR), showed a *reeler* phenotype (D’Arcangelo et al., 1999; Hiesberger et al., 1999; Trommsdorff et al., 1999). Mutants deficient in only one of these receptors showed phenotypes that were milder and also different from each other (Hack et al., 2007). Thus, while in *ApoER2* knockout mice late-generated neurons were unable to bypass early-generated cells like in the *reeler* mutant, mutants deficient in *VLDLR* displayed another feature of the *reeler* phenotype, the characteristic invasion of the MZ (Hack et al., 2007). Indeed, the two receptor types were found differently distributed in the cortex with the VLDLR receptor being mainly located in upper cortical layers (Hirota et al., 2014), suggesting that this receptor would be responsible for the stop signal function of Reelin (Hack et al., 2007). Besides these two lipoprotein receptors, cadherin-related neuronal receptors (CNRs; Senzaki et al., 1999) and alpha3beta1 integrins (Dulabon et al., 2000) were described to function as Reelin receptors. Binding of Reelin to ApoER2 and VLDLR was found to induce the phosphorylation of disabled 1 (Dab1; Howell et al., 1999; Benhayon et al., 2003), an adaptor protein binding to the intracellular domains of the lipoprotein receptors. Mutants deficient in Dab1 showed a *reeler*-like phenotype (Sheldon et al., 1997). The Reelin signaling cascade involves in addition src family kinases which phosphorylate Dab1, phosphatidylinositol 3-kinase (PI3K), and glycogen synthase kinase 3 beta (GSK3B), the latter kinase being known to phosphorylate Tau (Hiesberger et al., 1999). Hyperphosphorylation of Tau is a hallmark of Alzheimer’s disease, suggesting an involvement of the Reelin signaling cascade in the pathology of this disorder but further work is needed to obtain a clearer picture.

## Reelin Acts Upstream of Cofilin to Facilitate Migration

We previously found that Reelin induces phosphorylation (activation) of LIM kinase 1 (LIMK1), a kinase that in turn induces phosphorylation of cofilin (Chai et al., 2009). Cofilin is an actin-depolymerizing protein that is inactivated by its phosphorylation at serine3 (Arber et al., 1998; Yang et al., 1998). Since Reelin induces the phosphorylation of cofilin, one is tempted to conclude that Reelin counteracts cytoskeletal reorganization and in turn stabilizes the actin cytoskeleton in neuronal processes. In the present review article we will not discuss other molecules of the Reelin signaling cascade in detail but will focus on Reelin’s role in the stabilization of the actin cytoskeleton as an important prerequisite for the stability of the leading process which is needed for nuclear translocation during migratory activity. The reader is referred to several other review articles dealing in more detail with the various aspects of Reelin function (Rakic and Caviness, 1995; Curran and D’Arcangelo, 1998; Frotscher, 1998, 2010; Jossin et al., 2003; Tissir and Goffinet, 2003; Förster et al., 2006a,b; Cooper, 2008, 2013; Zhao and Frotscher, 2010; Chai and Frotscher, 2016).

**Figure 1:**
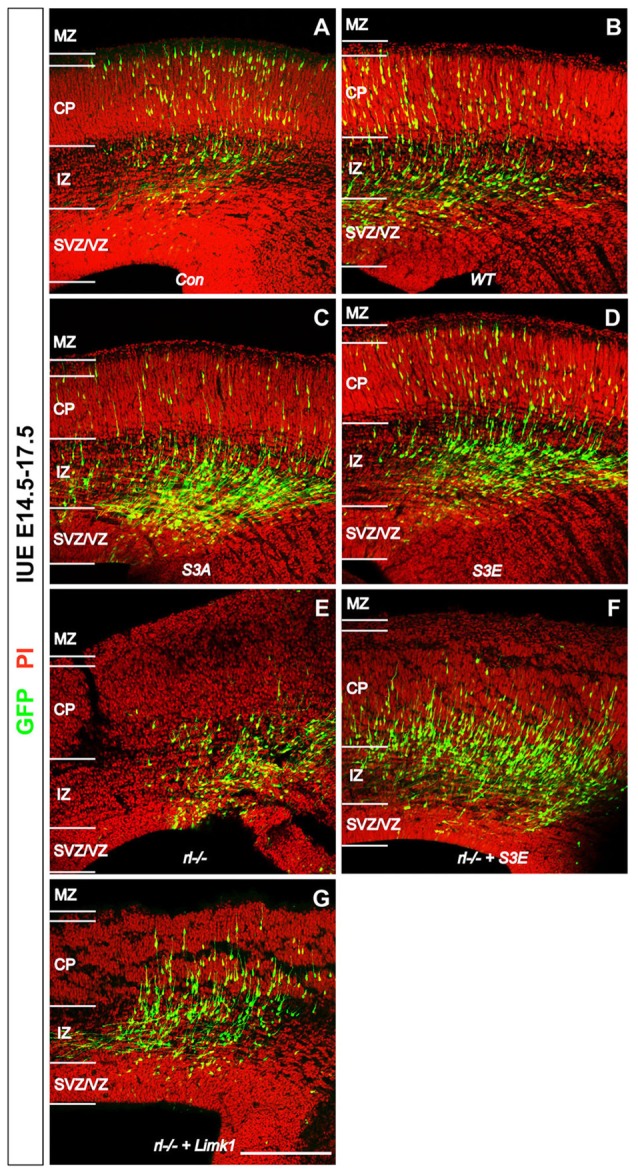
**Reelin-cofilin crosstalk during the migration of cortical neurons. (A,B)** In wild-type cortices (*Con*) and in cortices transfected with wild-type cofilin (*cofilin*^WT^), many GFP-positive cells have reached the cortical plate (CP) 3 days after *in utero* electroporation (IUE) on E 14.5. **(C,D)** In contrast, the majority of *cofilin*^S3A^- and *cofilin*^S3E^-transfected neurons have remained in the intermediate zone (IZ) and subventricular/ventricular zone (SVZ/VZ). **(E)** Similarly, very few cells have entered the CP in *reeler* slice cultures. **(F,G)** Slices from *reeler* embryos transfected with *cofilin*^S3E^ **(F)** or *LIM KINASE 1 (LIMK1)* **(G)** show partial rescue of the *reeler* phenotype with significantly more neurons invading the CP. MZ, marginal zone. Sections were counterstained with propidium iodide (PI). Scale bar: 100 μm (from Chai et al., 2016, reproduced with permission).

Some time ago Bellenchi et al. (2007) reported that conditional cofilin mutants showed migration defects reminiscent of the reeler phenotype. Chai et al. (2009) then demonstrated that the amounts of phosphorylated cofilin are significantly reduced in *reeler* and that application of recombinant Reelin to *reeler* tissue significantly increased the phosphorylation of cofilin, suggesting that Reelin-induced cofilin phosporylation is important for the normal lamination of the cerebral cortex. This hypothesis was recently confirmed by *in utero* electroporation (IUE) experiments (Chai et al., 2016). Transfection of newly born cortical neurons with a non-phosphorylatable form of cofilin (*cofilin*^S3A^) revealed a significant migration defect of the transfected cells, which remained near their site of origin in the subventricular zone (SVZ)—very much reminiscent of the *reeler* phenotype. Neurons transfected with a pseudophosphorylated form (*cofilin*^S3E^) also showed a migration defect. Remarkably, transfection of *reeler* embryos with *LIMK1* seemingly forced the phosphorylation of cofilin in these animals and resulted in a partial rescue of the migration defect in *reeler*, as did transfection with pseudophosphorylated *cofilin*^S3E^. These results on GFP-expressing neurons were obtained by IUE on embryonic day (E) 14.5, sacrificing the embryos on E17.5 and counterstaining sections of the cortex for propidium iodide (PI; Figure 1). Remarkably, neurons transfected with the cofilin mutants, similarly to GFP transfected *reeler* neurons, often showed leading processes oriented toward the ventricle. Collectively, these results suggest that Reelin-induced stabilization of the leading process, likely by the phosphorylation of cofilin, is an essential step in the proper orientation and migration directionality of late-generated cortical neurons.

## Reelin Acts on Both Neurons and Radial Glial Cells

During early stages of corticogenesis, newborn neurons find their way through the yet thin cortical wall. They only have to migrate from the ventricular zone (VZ)/SVZ to the deep cortical layers. As already pointed out, late-generated cortical neurons destined to superficial layers have to migrate for longer distances. This holds particularly true since the cortical plate (CP) and the intermediate zone (IZ) have become thicker at later developmental stages due to the continuous arrival of numerous neurons and the development of their dendritic and axonal processes. In order to traverse these larger distances successfully, late-generated neurons migrate along radial glial fibers, which span out between the ventricle and the pial surface, serving as a guiding scaffold for migrating cells (Rakic, 1972). Is Reelin involved in the correct orientation and targeting of radial glial fibers?

In a previous study, we were able to show that glial fibrillary acidic protein (GFAP)-positive processes of radial glial cells studied in a stripe choice assay with Reelin-containing stripes next to control stripes, started to branch as soon as they reached the Reelin stripes (Förster et al., 2002), suggesting that Reelin induces the branching of radial glial processes. Remarkably, when studying GFP-labeled radial glial fibers *in vivo*, they were found to branch as soon as they reached the Reelin-containing MZ (Chai et al., 2015). Next, we studied radial glial fibers in *reeler* mutant tissue and found that the number of branches arising from the main radial process was significantly reduced in *reeler* when compared to wild-type radial glial fibers (Chai et al., 2015). We concluded that Reelin induces the branching of radial glial fibers arriving at the MZ, thereby anchoring them to the cortical surface and allowing for the detachment of migrating neurons, which then finalize the migratory process by terminal translocation of the nucleus in the absence of a guiding radial fiber. Remarkably, branching of processes is associated with increased reorganization of the cytoskeleton, and future studies need to find out how Reelin in the MZ induces both branching and stabilization of the actin cytoskeleton by cofilin phosphorylation.

**Figure 2:**
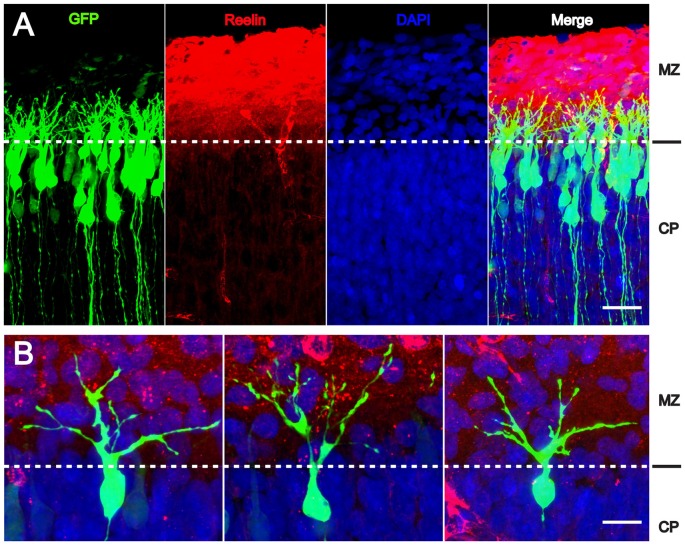
**Reelin-induced branching of the leading processes. (A,B)** The leading processes of migrating neurons start to branch as soon as they reach the MZ containing Reelin. Transfection of wild-type embryos with *CAG-GFP* on E 14.5; fixation on E 17.5. CP, cortical plate. Scale bars: 40 μm **(A)**; 15 μm **(B)** (modified from Chai et al., 2015, with permission).

**Figure 3:**
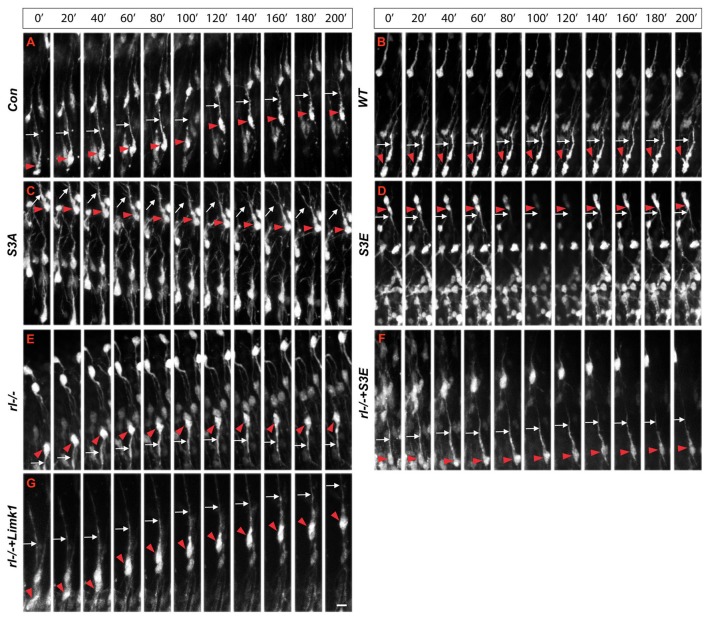
**Real-time microscopy of migrating neurons transfected with the different constructs. (A–G)** Following in IUE on E 14.5, slice cultures were prepared on E 17.5 and single neurons were monitored over a period of 200 min. Red arrowheads mark the cell bodies and white arrows the leading processes of migrating neurons. The MZ is at the top of the figures, the VZ at the bottom. Note aberrant leading processes pointing toward the VZ in *reeler* neurons (*rl*^−/−^) and neurons transfected with *cofilin*^S3E^. Neurons in *reeler* slices transfected with *LIMK1* or *cofilin*^S3E^ show a normal leading process oriented toward the MZ. Scale bar: 15 μm (from Chai et al., 2016, reproduced with permission).

Since radial glial cells are precursors of neurons, we hypothesized that also the leading processes of neurons would start to branch upon arrival at the MZ. Indeed, the leading processes gave rise to branches precisely upon arrival at the Reelin-containing MZ (Figure 2). In the mature animal these branches in layer I represent the apical tuft of cortical pyramidal cells. When studying branching of the leading processes by real-time microscopy, we noticed that migration by nuclear translocation was terminated as soon as the large nucleus arrived at the thin branches of the main shaft of the leading process, suggesting that mechanical obstruction might play a role in the migratory arrest of late-generated neurons (Chai et al., 2015; O’Dell et al., 2015). As a result, the MZ containing the thin branches of the leading processes is almost free of cell bodies. Accordingly, the stop signal function of Reelin would be two-fold: first, Reelin induces branching of the leading processes that cannot be entered by the translocating nucleus. Second, binding of Reelin to VLDLR receptors terminates migratory activity in wild-type neurons, but not in *reeler* neurons and in neurons deficient in VLDLR. Accordingly, many neurons in *VLDLR* knockout mice show “overmigration” into the MZ (Hack et al., 2007). In *reeler* mutants, Reelin-induced branching of radial glial fibers and of the leading processes of neurons is absent and neurons are not anchored to the MZ by these branches of the leading process. As a consequence, the leading processes do not remain attached to the cortical surface and point to various directions, often toward the VZ. We accordingly observed many neurons in *reeler* that did not migrate toward the cortical surface but in opposite direction toward the VZ. Similar observations were occasionally made in neurons transfected by IUE with the non-phosphorylatable form of cofilin (*cofilin*^S3A^) and more often in embryos transfected with *cofilin*^S3E^. These findings in particular underscore the significant role of Reelin-induced cofilin phosphorylation for the orientation of the leading process and directed migration of cortical neurons. Indeed, we have previously shown that the phosphorylated form of cofilin is much enriched in and near the MZ (Chai et al., 2009). It is remarkable to note in this context that transfection of *reeler* embryos with pseudophosphorylated *cofilin*^S3E^ or *LIMK1* partially rescued orientation of the leading processes and the migration defect in *reeler*. In order to study the migratory behavior of the living cells in some detail, IUE with the various constructs was performed on E 14.5 and slice cultures of neocortex were prepared 3 days later, when many neurons destined to superficial layers were migrating (Figure 3). Together, these findings indicate that Reelin plays an important role in the proper orientation and stabilization of the leading process, allowing for directed migration towards the cortical surface. Moreover, proper orientation of the leading process and its attachment to the MZ provides the tension required for nuclear translocation and the successful bypassing of early generated deep-layer neurons.

We were impressed to find such significant migration defects when transfecting wild-type embryos with *cofilin^S3A^* and *cofilin*^S3E^ and to observe partial rescue of the *reeler* phenotype following transfection with *LIMK1*—although many other molecular players such as molecules of the tubulin cytoskeleton (e.g., Meseke et al., 2013; Förster, 2014), proneural transcription factors such as Rnd proteins (Pacary et al., 2011), and CLASP2 (Dillon et al., 2017) are also known to control the cytoskeleton of neuronal cells and extension and orientation of the leading process, migration by nuclear translocation associated with a myosin II-dependent flow of actin filaments (He et al., 2010), and eventually layer formation in the cerebral cortex.

## Author Contributions

MF developed the concept of this review article and wrote the manuscript. SZ, SW and XC performed the majority of the experiments reviewed in this article and contributed to manuscript writing and editing.

## Conflict of Interest Statement

The authors declare that the research was conducted in the absence of any commercial or financial relationships that could be construed as a potential conflict of interest.
